# Thermal and Electrochemical Properties of Ionic Liquids Bearing Allyl Group with Sulfonate-Based Anions—Application Potential in Epoxy Resin Curing Process

**DOI:** 10.3390/molecules28020709

**Published:** 2023-01-10

**Authors:** Andrea Szpecht, Dawid Zielinski, Maciej Galinski, Marcin Smiglak

**Affiliations:** 1 Poznan Science and Technology Park, Adam Mickiewicz University Foundation, 61-612 Poznań, Poland; 2 Faculty of Chemistry, Adam Mickiewicz University, 61-614 Poznań, Poland; 3 Institute of Chemistry and Technical Electrochemistry, Poznan University of Technology, 60-965 Poznań, Poland

**Keywords:** ionic liquids, allyl-based ionic liquids, thermal properties, conductivity, electrochemical window, epoxy resins

## Abstract

Sulfonate-based ionic liquids (ILs) with allyl-containing cations have been previously obtained by us, however, the present study aims to investigate the thermal, electrochemical and curing properties of these ILs. To determine the temperature range in which ionic liquid maintains a liquid state, thermal properties must be examined using Differential Scanning Calorimetry (DSC) and Thermogravimetric Analysis (TGA). Melting, cold crystallization and glass transition temperatures are discussed, as well as decomposition temperatures for imidazolium- and pyridinium-based ionic liquids. The conductivity and electrochemical stability ranges were studied in order to investigate their potential applicability as electrolytes. Finally, the potential of triflate-based ILs as polymerization initiators for epoxy resins was proven.

## 1. Introduction

In the search of new chemicals that can be seen as replacements for traditional solvents, ionic liquids (ILs) emerged at the beginning of 2000 as promising solution [1]. Since then, it appears that they have been tested for almost every application possible, and in some cases they have already been implemented as a better solution for certain companies [2]. ILs present appealing properties, such as low volatility, high thermal stability and high ionic conductivity; however, the most important feature of ILs is their tunability for specific applications [3]. The ionic structures can be limitless, therefore, they have been one of the most studied subjects in green chemistry for the last few years [4].

The attractiveness of ILs to ever-expanding research areas is dictated by the low volatility of these compounds and their non-flammability, high chemical inertness, high ionic conductivity and thermal stability [5]. Possibilities of tuning the properties of ILs by changes in ion structures are endless, and thus they are used as solvents designed for a special purpose [3]. Ionic liquids are considered as green solvents, which cannot be said about standard organic solvents used in organic synthesis. In addition, they can be recycled, and they are immiscible with most organic solvents (easy separation) [1].

Further modification of cation structure can offer pathways to more applications [6,7]. Compounds having an unsaturated group in their structure are a special group of ionic liquids. The introduction of a group with a terminal double bond, e.g., allyl [8], 4-vinylbenzyl [9], makes them multifunctional ILs, with the possibility of further transformations. The main reaction which such monomers are subjected to are polymerization reactions, e.g., Atom Transfer Radical Polymerization (ATRP) [10] and Reversible Addition Fragmentation Chain-Transfer polymerization (RAFT) [11]. Such modifications allow to obtain Polymerized Ionic Liquids (PILs) [12]. ILs containing a polymerizable group in the cation structure can serve as a kind of building block of ionic polymers [13], the potential applications of which include, for example, CO_2_ capture [14], adsorption of metal ions [15], electrochemical devices [16] or ionogel preparation [17].

Additionally, anions can also be tailored to the desired application of ionic liquids [18]. For a special group of anions we can consider sulfonate-based anions, such as methylsulfate (MeSO_4_), methanesulfonate (MeSO_3_), triflate (OTf) and tosylate (OTs) [19]. In our previous work, we demonstrated a simple and extremely effective method of the synthesis of ILs with the abovementioned anions [20]. The main advantage of the proposed metathesis reaction is the ability to obtain ionic liquids with very high purities, with no or negligible halide impurities. Furthermore, the described method allows to obtain compounds with no restrictions to the cation structure in comparison to the widely used synthetic route, where the tertiary amine undergoes a quaternization reaction with methylated ester of sulfonate-based acid. As a result, the obtained salt always has an alkyl group (methyl or ethyl) in the cation structure. This is quite important disadvantage, because it limits the possibilities of ionic liquids and their potential applications. In electrochemistry, purity is the most important feature of an electrolyte [21]. It has been previously shown that ILs with trifluoromethanesulfonate, methanesulfonate, methylsulfate and *p*-toluenesulfonate anions have found their dominant application in the field of electrochemistry [22]. They are used as electrolytes in (i) novel energy storage devices [23]; (ii) batteries as gels [24]; (iii) electrochemical cells with salt-based metal [25]; (iv) supercapacitors [26].

Moreover, ionic liquids have been found to be a new wave of hardeners for epoxy resin polymerization [27]. The typically used amines or anhydrides present many drawbacks for modern applications [28], where ILs provide better solutions [29]. Firstly, the mixture of ionic liquid/epoxy resin can be stored at room temperature for a long time because the polymerization process starts only at elevated temperature. Additionally, the quantity of IL needed for the successful hardening process is much lower than the abovementioned compounds, at around 5% weight (or in many cases even less) [30]. The vast majority of scientific research has focused on imidazolium-based ionic liquids with dicyanamide (DCA) and acetate anions [31,32,33], because of their liquid state at room temperature (which makes it easy for mixing with resins), their aromatic imidazolium ring and highly active anions, which determine the peak polymerization temperature for the process. The effect of different anions, such as tetrafluoroborate (BF_4_) [34], have also been studied. On the other hand, phosphonium-based ILs are promising new hardeners for commercially available epoxy resins, as well as DGEBA molecules, while simultaneously being flame retardants [35,36].

Herein, we present the physicochemical properties of 12 ionic liquids based on three aromatic rings (two imidazolium and one pyridinium) with an allyl group in the cation structure and sulfonate-based anions (OTf, OTs, MeSO_3_ and MeSO_4_). The present study showcases the electrochemical properties of high-purity ionic liquids, alongside their thermal properties. Finally, all ionic liquids were employed in the polymerization process of epoxy resin.

## 2. Results and Discussion

The synthesis of eight ionic liquids discussed in this paper has already been reported by us [20]. We showcased the versatile method of obtaining ILs with sulfonate-based anions, with high purities and high atom economies. All ionic liquids were new in the literature, and to this day there is no report on their physiochemical properties. The only data that are available originate from our paper, and it was limited to chromatographic data and NMR spectroscopy. Synthesis of the four ionic liquids that were not included in the previous article follows the same preparation method. Experimental information can be found in the Supplementary Material alongside NMR spectra (S1–S4).

### 2.1. Differential Scanning Calorimetry Analysis of Allyl-Based Ionic Liquids

All ionic liquids presented in this research were characterized by Differential Scanning Calorimetry (DSC) to determine the presence of any thermal transitions. The melting temperature for ionic liquids represents the lower limit of the liquid gap and alongside decomposition temperature defines the temperature range in which ILs are liquid and therefore can be used as a solvent.

**Figure 1:**
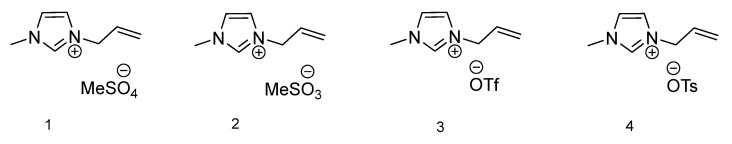
Structures of ionic liquids based on 1-allyl-3-methylimidazolium cation.

The first group of ionic liquids comprises of 1-methylimidazole ring equipped with one unsaturated group (allyl) and four different anions. Figure 1 presents the chemical structures of the abovementioned ILs. For ionic liquid with methylsulfate anion **1**, only glass transition temperature at −54.55 °C is observable. This can be explained by the liquid state of this compound at room temperature. For the second IL with methanesulfonate anion **2**, similarly only T_g_ is observable at −39.87 °C. Compound **3**, 1-allyl-3-methylimidazolium triflate, presents no thermal transitions in the considered temperature range. The fourth ionic liquid from this group is characterized by the presence of a tosylate anion (**4**), and the only existing phase transition is T_g_ at −37.41 °C. All four ionic liquids with a 1-allyl-3-methylimidazolium-based cation are liquid at room temperature, therefore display neither melting or crystallization temperature. For compounds **1**, **2** and **4**, only glass transition temperature is observed. The vitrification process occurs, which transforms the substance into a glass state upon cooling [37]. The glass transition temperature is the midpoint of a small heat capacity change upon heating from the glassy solid to a liquid, where below T_g_ materials are glassy and above T_g_ are liquid [38].

**Figure 2:**
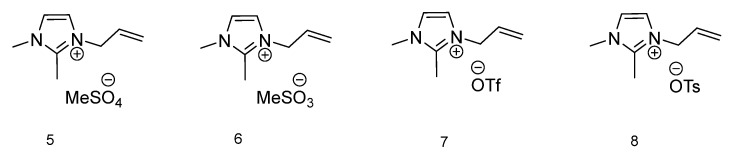
Structures of ionic liquids based on 1-allyl-2,3-dimethylimidazolium cation.

To compare the effect of H-bonding interactions between the ions, the thermal phase transitions for 1-allyl-2,3-dimethylimidazolium-based ionic liquids (Figure 2) were measured. In four ionic liquids with an imidazolium ring, the C2–H proton is replaced by the methyl group. In Table 1 thermal transitions for all 12 ILs are presented. For compounds **5** and **6** with methylsulfate and methanesulfonate anions respectively, only one thermal transition is observable, and it is T_g_ for IL **5** (−63.26 °C). Ionic liquids **7** and **8** both have in their anion structure a CF_3_ group. The IL with a trifluoromethanesulfonate anion presents with two phase transitions, typical for ionic liquids, with a melting temperature at 51.20 °C and cold crystallization at 29.71 °C. The compound with the most sterically demanding anion **8**, *p*-toluenesulfonate, has a melting temperature higher than the one recognized for the possibility to be affiliated with ionic liquids (100 °C). However, since the 100 °C temperature is contractual, the 126.06 °C melting temperature of compound **8** still classifies 1-allyl-2,3-dimethylimidazolium tosylate as an ionic liquid. Additionally, the cold crystallization temperature is also observable at 47.72 °C. With only two ionic liquids displaying melting temperatures, it can be concluded that their solid and liquid phases are in equilibrium [39].

**Figure 3:**
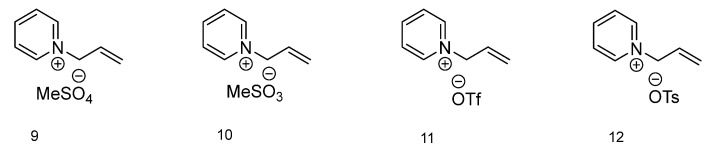
Structures of ionic liquids based on 1-allylpyridinium cation.

The final group of ionic liquids discussed within this paper has a different aromatic amine as a base for cation formation (Figure 3). Compound with methylsulfate as an anion (**9**) has no thermal transitions present on the DSC thermogram. 1-Allylpirydinium methanesulfonate (**10**) has a melting (76.03 °C) and cold crystallization (1.75 °C) as well as a glass transition temperature (–57.19 °C). The ionic liquid with the triflate anion (**11**) on the DSC chart is characterized by a melting temperature at 1.34 °C and a cold crystallization temp. at −15.46 °C. Ionic liquid **12**, with a tosylate anion, has similar temperature values as IL **10**, with a melting temp. at 96.60 °C, cold crystallization temp. at 4.16 °C and glass transition temp. at –33.70 °C.

For all ionic liquids discussed within this paper, there are no data available for which to compare the obtained results. However, thermal transitions for each cation paired with either chloride or bis(trifluoromethanesulfonyl)amide have been previously determined by our group, and the results can be found in Table 2. The halide salt of 1-allyl-3-methylimidazolium cation (**1** [Cl]) has only a glass transition temperature at −65.8 °C, and the bis(trifluoromethane)sulfonimide salt (**1** [NTf_2_]) presents with a melting temperature at −10 °C [8]. 1-Allyl-2,3-dimethylimidazolium chloride (**5** [Cl]) is characterized by the melting temperature at 116.6 °C, whilst the NTf_2_ analogue (**5** [NTf_2_]) had no observable phase transitions in the considered temperature range [8]. Finally, the chloride salt of the 1-allylpirydinium cation (**9** [Cl]) has a melting temperature at 109.8 °C, and 1-allylpirydinium bis(trifluoromethanesulfonyl)amide (**9** [NTf_2_]) has a T_m_ value of −19.6 °C.

The presented data above show that the ionic liquids based on the 1-allyl-3-methylimidazolium cation present no melting or crystallization affinity, which occurs in both other cation groups. The most phase transition resistant are the compounds with methylsulfate as an anion. The highest melting temperatures were displayed in the two ionic liquids with a *p*-toluenesulfonate anion (**8** and **12**), whereas the lowest were the triflate-based ILs (**7** and **11**). This can be explained by the sufficient steric hindrance that disrupts the packaging efficiency, furthermore minimizing the H-bonding interactions between ions [40].

### 2.2. Thermogravimetric Analysis of Allyl-Based Ionic Liquids

To determine the thermal stability of the ionic liquids, Thermogravimetric Analysis was employed, as it is the most used tool for the determination of such properties. All obtained results are presented in Table 3.

For ionic liquids with one methyl group present in the imidazole ring (**1**–**4**), the onset of decomposition temperatures range from 208.08 °C to 330.50 °C. 1-Allyl-3-methylimidazolium tosylate (**4**) has the highest T_onset_ value, while 1-allyl-3-methylimidazolium methylsulfate (**1**) has the lowest value. Compounds **1** and **3** are also characterized by second decomposition steps at 309.92 °C and 390.48 °C, respectively. Subsequently, employing the same method, the thermal stabilities of 1-allyl-2,3-dimethylimidazolium-based ILs (**5**–**8**) were determined, with the T_onset_ ranging from 225.33 °C to 341.81 °C. In contrast to the other imidazolium-based ionic liquids, for this group, the compound with trifluoromethanesulfonate anion (**7**) has the lowest onset decomposition temperature, while the highest one is attributed to the IL with methanesulfonate as an anion (**6**), but not far behind is 1-allyl-2,3-dimethylimidazolium tosylate (**8**) with T_onset_ at 337.03 °C. Lastly, the compounds with pyridine as an amine base for quaternization reactions (**9**–**12**) were studied, and the decomposition temperature range is from 234.36 °C to 288.30 °C. Likewise, 1-allylpirydinium tosylate (**12**) has the highest onset decomposition temperature of 288.30 °C, but the lowest is attributed to the triflate (**11**) analogue at 234.36 °C.

Based on the obtained results, it should be noted that ILs based on pyridine are less stable compared to those based on an imidazole ring, which is in agreement with the previous literature reports [41]. Furthermore, the replacement of the acidic C2 hydrogen in 1-methylimidazole causes the increase in the thermal stability, except in triflate-based ionic liquids, where the onset decomposition temperature decreased by 9.64 °C. Compounds **1**, **3**, **7**, **10** and **11** present second decomposition steps, with all three ionic liquids with trifluoromethanesulfonate as their anion. Moreover, it is well known that the thermal stability of ionic liquids depends heavily on the type of anion [42]. The most resistant to high temperatures are ionic liquids **4**, **8** and **12**, which possess tosylate anions in their structure. This can be explained by the poor coordinating nature of the *p*-toluenesulfonate anion, as well as its size. The degradation temperature is higher for ionic liquids with anions containing sulfonyl groups and lower for halide anions, which are highly coordinating moieties [43]. Additionally, the thermal stability of ILs is influenced by the water affinity, where the higher values of decomposition temperatures are characteristic for hydrophobic anions [44].

### 2.3. Electrochemical Properties of Allyl-Based Ionic Liquids

As ionic liquids can be used in many electrochemical applications, the best way to assess their electrochemical properties is to determine the width of the electrochemical window, referred to as the range in which ionic liquid is electrochemically inert, expressed in volts [45]. From the presented compounds in this research only seven are liquid at room temperature (RTILs), and their conductivity and electrochemical stability values are presented in Table 4.

Every ionic liquid with a 1-allyl-3-methylimidazolium-based cation (**1**–**4**) is liquid, and therefore the conductivity measurements were possible. From the remaining three compounds, two of them have methylsulfate as an anion (**5** and **9**), whereas the last one is 1-allylpyridinium triflate (**11**). The lowest conductivities were displayed by 1-allyl-3-methylimidazolium tosylate at 0.26 mS/cm (**4**) and 1-allyl-2,3-dimethylimidazolium methylsulfate at 0.28 mS/cm (**5**). On the other hand, the highest value was presented by the 1-allylpiridynium trifluoromethanesulfonate (**11**) ionic liquid. The other five ILs have conductivities in the range of 1.48 to 4.51 mS/cm. Since ionic conductivity is correlated to the viscosity of ionic liquid, it explains the low values for ILs **4** and **5**. However, other factors also need to be considered for having an impact on the conductivity values, namely density, ion size or charge delocalization [46].

**Figure 4:**
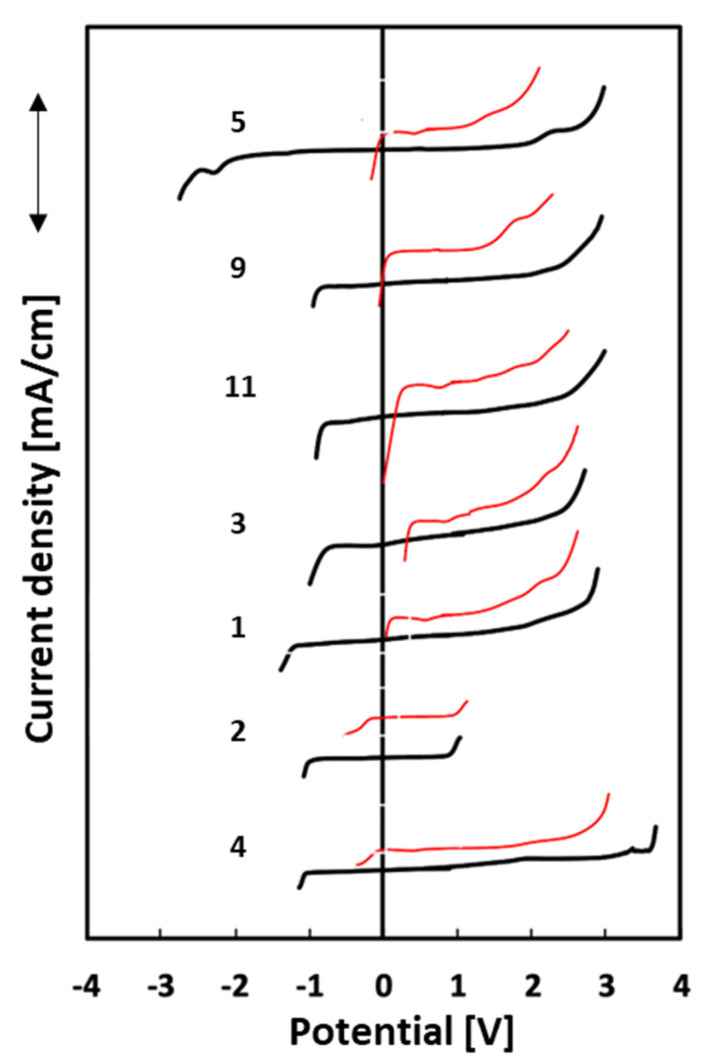
Electrochemical stability ranges for 7 ionic liquids: platinum electrode (Pt)—red lines, glass-like carbon electrode (GC)—black lines.

The electrochemical stability window (ESW) was also determined using two different electrodes, platinum, and glassy carbon. The lowest ESW value is observed for IL **2** and the highest for IL **4**, when the experiments were conducted with a Pt electrode. The range of all obtained results is relatively small, from 1.1 V to 2.8 V. Both ionic liquids have 1-allyl-3-methylimidazolium as a cation, but the first one is characterized by the methylsulfonate anion and the second one by the tosylate anion. On the other hand, the electrochemical stability ranges for the glassy carbon electrode presented the best result for 1-allyl-2,3-dimethylimidazolium methylsulfate at 5.8 V (**5**) and the lowest for 1-allyl-3-methylimidazolium methanesulfonate at 1.9 V (**2**). In every case, the range of electrochemical stability is notably smaller on the platinum electrode than on the glassy carbon electrode. It is a well-known phenomenon that Pt is a better catalyst, characterized by low values of decomposition overpotentials [47]. The results of the electrochemical stability ranges are shown in Figure 4.

It should be noted that compound **5**, while having the widest electrochemical stability range on the GC electrode, is also characterized by the broad side of negative potentials. Cations generally determine the cathodic stability limits, while the anodic limit is determined by anions [48]. Nonetheless, they may influence each other. The abovementioned case may be explained by the presence of the methyl group in the C2 position in the imidazole ring. 1-Allyl-3-methylimidazolium tosylate (**4**) has the widest ESW on the side of positive potentials, not only for the glassy carbon electrode but also for the platinum one. From the obtained results, it can be concluded that ionic liquids based on an imidazole ring have bigger electrochemical stability ranges than ILs based on pyridine. The one exception is the 1-allyl-3-methylimidazolium methanesulfonate (**2**) ionic liquid, which has the narrowest ranges for both electrodes. Two compounds (**4** and **5**) with the lowest conductivity values have the widest electrochemical stability ranges for Pt and GC electrodes. 

### 2.4. Curing Properties of Allyl-Based Ionic Liquids

Ionic liquids have been found to be an interesting alternative to the commercially available epoxy resin hardeners. The final objective of presented research was to examine the obtained ionic liquids for their potential hardening activity towards the Bisphenol A diglycidyl ether (DGEBA (M_n_ = 340 g/mol)) molecule, which is a constituent of epoxy resins. The curing activity of sulfonate-based ionic liquids has been examined using DSC measurements. From all the compounds presented in this paper, only trifluoromethanesulfonate-based ILs (**3**, **7** and **11**) displayed any polymerization capability towards the Bisphenol A diglycidyl ether molecule. Table 5 presents characteristic temperatures and enthalpies for triflate-based ionic liquids.

The onset temperatures are within the range of 149.52–176.70 °C, while the end temperature points of polymerization are from 173.58 °C to 231.89 °C. However, the most important temperature value seems to be the temperature at which the polymerization reaches its peak (T_max_). 1-Allyl-3-methylmidazolium triflate (**3**) and 1-allylpirydinium triflate (**11**) have very similar temperature values across all measured points. On the other hand, compound **7** has the lowest T_onset_ value but the highest T_max_ and T_endset_ values. All ionic liquids were used at 2% wt, hence the reaction enthalpies can be compared. The highest enthalpy presented ionic liquid based on the 1-allylpyridine cation (**11**), at 554.58 J/g. The triflate anion is the initiator for anionic homopolymerization in all cases, and the cation structure also has an impact on the exothermic value of the reaction. 

**Figure 5:**
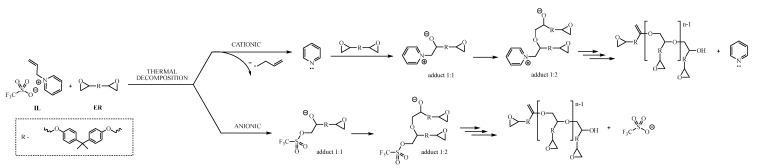
Proposed pathway of epoxy resin polymerization, initiated by triflate-based IL.

Both pyridine and imidazole, in the form of pure amines, can initiate the polymerization of epoxy resins. Each of them cures the resin according to a different mechanism, but in the case of the tested ionic liquids, the cationic curing initiation pathway involving the cation structure of the ionic liquid is dominated by the anion pathway involving the OTf anion. The energy of the process expressed in the form of enthalpy of thermal transformation is so high that the triflate anion dominates the curing process, which has a much greater affinity for the DGEBA molecule than cations of ionic liquids. Moreover, it shows the great potential of sulfur anions in applications as curing initiators. On the other hand, the process of epoxy polymerization initiated by the triflate is a rapid and high-energy process, which forces the polymerization process to be carefully controlled because exceeding the thermal peak temperature too quickly may cause the curing process to proceed uncontrollably and lead to excessive overheating of the system, and consequently poor parameters of the final, hardened material. Figure 5 proposes a pathway for the polymerization reaction between epoxy resin and 1-allylpyridinium triflate (**11**).

## 3. Materials and Methods

Ionic liquids used in this paper were previously obtained by us. Other chemicals were of high purity and purchased form Merck and used as received.

Melting points (T_m_), crystallization temperatures (T_c_) and glass transition states (T_g_) for pure ionic liquids and/or organic salts were determined using Differential Scanning Calorimetry (DSC). The procedure was as follows: (i) 1st heating cycle from 25 °C to 130 °C; (ii) 1st cooling cycle from 130 °C to −80 °C; (iii) 2nd heating cycle from −80 °C to 200 °C; (iv) 2nd cooling cycle from 200 °C to 25 °C. The heating and cooling rate was 10 °C/min.

The polymerization process for the ionic liquid/epoxy resin systems was determined using DSC. All the samples were freshly prepared by mixing ionic liquids (0.1 g) with epoxy resin (5 g) in a glass vial. Each sample was approximately 10 mg. The procedure was as follows: (i) 1st heating cycle from 25 °C to 250 °C; (ii) 1st cooling from 250 °C to 25 °C. The heating and cooling rate was 10 °C/min.

All the DSC data were collected by the same calorimeter, a Mettler-Toledo DSC 1 STARe System differential scanning calorimeter, cooled with a Huber TC100 immersion cooler. All data were collected at atmospheric pressure, with nitrogen as a purge gas, and an empty sample pan as the reference.

Thermal stabilities were investigated using a TA Instrument TGA Q50 thermogravimetric analyzer, precise to within 0.1 °C in temperature and to within 0.01% in weight. The TGA experiments were conducted under nitrogen atmosphere and measured in the dynamic heating regime. Samples between 5 and 10 mg were heated from 25 °C to 600 °C with a heating rate of 5 °C/min with a 10 min isotherm at 85 °C. This isotherm step was intended to remove any remaining water present in the samples. Decomposition temperatures reported for all materials were established as the onset temperature for decomposition of the first 5% of the sample (T_5%onset_), as the regular onset temperature for decomposition (T_onset_) for the whole sample and as the onset temperature of the second decomposition step (T_1_). 

Conductivity measurements were conducted by placing ca. 1 mL of the IL into the conductivity cell. The conductivity cell was built of two parallel platinum plates of ca. 5 mm diameter embedded in a glass container with a water jacket. The Electrochemical Impedance Spectroscopy (EIS) technique was used to determine electrolytic conductivity. The spectra were measured in the frequency range from 100 kHz to 1 mHz, at a voltage amplitude of 10 mV. From EIS spectra, the resistance values were determined and used to calculate the specific conductivity of the ionic liquids. The conductivity cell was calibrated by the commercial 0.01 M KCl calibrating solution (Hydromet, Gliwice, Poland). The electrolytic cell constant was 0.188 cm^−1^.

Determination of the ranges of electrochemical stability of ionic liquids was performed by measuring linear voltammetry in a 3-electrode system. The test electrodes were a platinum electrode (disc with an area of 0.202 cm^2^) and (alternatively) a glassy carbon electrode (area of 0.0785 cm^2^). The reference electrode was a cryptic electrode (Ag + 222 in acetonitrile), while the counter electrode was a platinum plate with an area of about 2 cm^2^. The measurement consisted of a linear change in potential with a shift speed of 5 mV/s until the specified current value (1 mA/cm^2^) was exceeded, which is considered to be the value at which redox reactions (electrolyte decomposition) occur.

## 4. Conclusions

The series of 12 ionic liquids containing allyl groups in the cation structure paired with sulfonate-based anions were tested for their thermal, electrochemical and curing properties. Until now, there were no research data available on the properties of research compounds. From three different cation groups with four different anions, the most thermally inert are ionic liquids with methylsulfate as an anion, presenting only glass transition temperatures in two out of three cases. Furthermore, 1-allyl-3-methylimidazolium-based compounds also are the most resistant to phase transitions (only T_g_ for ILs **1**, **2** and **4**). Ionic liquids with tosylate anions displayed the highest melting temperature values, and the lowest was attributed to the triflate-containing compounds. The most thermally stable are ionic liquids **4**, **8** and **12** with a *p*-toluenesulfonate anion, because of its poor coordinating nature and big size. In addition, pyridine-based ILs are less resistant to high temperatures than imidazolium-based ILs. For ionic liquids with methylsulfate and methanesulfonate anions, the obtained results point to the lowest thermal stabilities, which are most likely caused by the nature of the leaving group and the structure of the acid that anions originate from. The same explanation can be transferred to DSC results, where ILs with an MeSO_4_ anion display only glass transition temperatures (or in one case no phase transition), whereas the MeSO_3_-based ionic liquids have very similar properties, with one exemption, 1-allylpyridinium methanesulfonate (**10**), which is solid at room temperature and presents on the DSC chart a melting temperature, crystallization temperature and glass transition temperature. This anomaly can be attributed to the nature of the pyridine cation. The lowest conductivity values are exhibited by compounds **4** and **5**, which are in correlation to their high viscosity. The best electrochemical stability on the platinum and glassy carbon electrodes was shown by 1-allyl-3-methylimidazolium tosylate (**4**) and **1**-allyl-2,3-dimethylimidazolium methylsulfate (**5**), respectively. All ESW values were higher for the GC electrode. Imidazolium-based ionic liquids have better electrochemical properties than pyridine-based. Despite their low conductivities, compounds **4** and **5** have the widest electrochemical stability ranges for both electrodes. Finally, triflate-based ionic liquids were found to be exhibit curing activity towards the constituent molecule of epoxy resins (DGEBA). All three ILs presented similar temperature values for onset, max and endset polymerizations. In contrast, the highest exothermic energy was characteristic for 1-allylpyridine triflate (**11**). In conclusion, the obtained results for the thermal and electrochemical properties of the sulfonate-based ionic liquids are reported for the first time in the literature. This knowledge can enhance the possibilities of further investigation of the researched ionic liquids in possible applications, such as battery or supercapacitor electrolytes, as well as a new class of epoxy resin hardeners. 

## Figures and Tables

**Table 1:** Thermal transitions obtained with DSC measurements for 12 ionic liquids.

IL Number	T_m_ (°C)	T_c_ (°C)	T_g_ (°C)
1	-	-	−54.55
2	-	-	−39.87
3	-	-	-
4	-	-	−37.41
5	-	-	−63.26
6	-	-	-
7	51.20	29.71	-
8	126.06	47.72	-
9	-	-	-
10	73.06	1.75	−57.19
11	1.34	−15.46	-
12	96.90	4.16	−33.70

T_m_—melting temperature; T_c_—crystallization temperature; T_g_—glass transition temperature.

**Table 2:** Thermal transitions for chlorides and bis(trifluoromethanesulfonyl)amides of three examined cation groups.

IL Number	T_m_ (°C)	T_c_ (°C)	T_g_ (°C)
1 [Cl]	-	-	−65.8
1 [NTf_2_]	−10	-	-
5 [Cl]	116.6	-	-
5 [NTf_2_]	-	-	-
9 [Cl]	109.8	-	-
9 [NTf_2_]	−19.6	-	-

T_m_—melting temperature; T_c_—crystallization temperature; T_g_—glass transition temperature.

**Table 3:** Thermal decomposition temperatures obtained with TGA measurements for all ionic liquids presented in this study.

IL Number	T_onset_ (°C)	T_onset5%_ (°C)	T_1_ (°C)
1	208.08	179.38	309.92
2	328.11	300.80	-
3	234.97	191.80	390.48
4	330.50	316.45	-
5	290.83	-	-
6	341.81	316.81	-
7	225.33	174.71	404.68
8	337.03	315.11	-
9	276.02	240.97	-
10	255.20	222.44	307.54
11	234.36	202.22	351.58
12	288.30	264.25	-

T_onset_—onset temperature for decomposition; T_onset5%_—onset temperature of the decomposition of the first 5% of the sample; T_1_—onset temperature of the second decomposition step.

**Table 4:** Conductivity values and electrochemical stability ranges for the RTILs.

IL Number	Conductivity (mS/cm)	Electrochemical Stability Range (V)	
		Pt	GC
1	1.48	2.0	4.0
2	1.95	1.1	1.9
3	4.51	2.6	3.8
4	0.26	2.8	4.6
5	0.28	1.8	5.8
9	2.34	1.3	3.5
11	5.62	1.7	3.6

Pt—platinum electrode; GC—glassy carbon electrode.

**Table 5:** Curing properties of triflate-based ionic liquids.

IL Number	T_onset_ (°C)	T_max_ (°C)	T_endset_ (°C)	∆H (J/g)
3	176.70	179.01	179.25	380.27
7	149.52	216.85	231.89	480.42
11	168.35	172.84	173.58	554.58

T_onset_—onset temperature of polymerization; T_max_—maximum temperature peak of polymerization; T_endset_—end temperature point of polymerization; ∆H—enthalpy of polymerization.

## Data Availability

The original data presented in this study are from the authors upon request.

## Supplementary Materials

The following supporting information can be downloaded at: https://www.mdpi.com/article/10.3390/molecules28020709/s1, NMR analysis and spectra of compounds **1**–**4** (S1–S4).

## References

[1] Earle M.J., Seddon K.R. (2000). Ionic Liquids. Green Solvents for the Future. Pure Appl. Chem..

[2] Wineinger H.B., Kelly A., Shamshina J.L., Rogers R.D. (2020). Farmed Jumbo Shrimp Molts: An Ionic Liquid Strategy to Increase Chitin Yield per Animal While Controlling Molecular Weight. Green Chem..

[3] Hallett J.P., Welton T. (2011). Room-Temperature Ionic Liquids: Solvents for Synthesis and Catalysis. 2. Chem. Rev..

[4] Quintana A.A., Sztapka A.M., Ebinuma V.D.C.S., Agatemor C. (2022). Enabling Sustainable Chemistry with Ionic Liquids and Deep Eutectic Solvents: A Fad or the Future?. Angew. Chem. Int. Ed..

[5] Chiappe C., Pieraccini D. (2005). Ionic Liquids: Solvent Properties and Organic Reactivity. J. Phys. Org. Chem..

[6] Philippi F., Welton T. (2021). Targeted Modifications in Ionic Liquids—From Understanding to Design. Phys. Chem. Chem. Phys..

[7] Greer A.J., Jacquemin J., Hardacre C. (2020). Industrial Applications of Ionic Liquids. Molecules.

[8] Zajac A., Szpecht A., Szymanska A., Zielinski D., Stolarska O., Smiglak M., Maciejewski H. (2020). Synthesis and Characterization of Nitrogen-Based Ionic Liquids Bearing Allyl Groups and Examples of Their Application. New J. Chem..

[9] Zajac A., Szpecht A., Zielinski D., Rola K., Hoppe J., Komorowska K., Smiglak M. (2019). Synthesis and Characterization of Potentially Polymerizable Amine-Derived Ionic Liquids Bearing 4-Vinylbenzyl Group. J. Mol. Liq..

[10] He H., Luebke D., Nulwala H., Matyjaszewski K. (2014). Synthesis of Poly(Ionic Liquid)s by Atom Transfer Radical Polymerization with Ppm of Cu Catalyst. Macromolecules.

[11] Nakabayashi K., Sato Y., Isawa Y., Lo C.-T., Mori H. (2017). Ionic Conductivity and Assembled Structures of Imidazolium Salt-Based Block Copolymers with Thermoresponsive Segments. Polymers.

[12] Maksym P., Tarnacka M., Bielas R., Hachuła B., Zajac A., Szpecht A., Smiglak M., Kaminski K., Paluch M. (2020). Structure-Property Relationships of Tailored Imidazolium- and Pyrrolidinium-Based Poly(Ionic Liquid)s. Solid-like vs. Gel-like Systems. Polymer.

[13] Green O., Grubjesic S., Lee S., Firestone M.A. (2009). The Design of Polymeric Ionic Liquids for the Preparation of Functional Materials. Polym. Rev..

[14] Zulfiqar S., Ilyas Sarwar M., Mecerreyes D. (2015). Polymeric Ionic Liquids for CO_2_ Capture and Separation: Potential, Progress and Challenges. Polym. Chem..

[15] Wieszczycka K., Filipowiak K., Wojciechowska I., Aksamitowski P. (2020). Novel Ionic Liquid-Modified Polymers for Highly Effective Adsorption of Heavy Metals Ions. Sep. Purif. Technol..

[16] Mecerreyes D. (2011). Polymeric Ionic Liquids: Broadening the Properties and Applications of Polyelectrolytes. Prog. Polym. Sci..

[17] Lewandowska A., Gajewski P., Szcześniak K., Fojud Z., Robakowska M., Skrzypczak A., Voelkel A., Marcinkowska A. (2022). Thiol–Ene Ionogels Based on Polymerizable Imidazolium Ionic Liquids. Polym. Chem..

[18] Seki S., Kobayashi T., Kobayashi Y., Takei K., Miyashiro H., Hayamizu K., Tsuzuki S., Mitsugi T., Umebayashi Y. (2010). Effects of Cation and Anion on Physical Properties of Room-Temperature Ionic Liquids. J. Mol. Liq..

[19] Lemus J., Neves C.M.S.S., Marques C.F.C., Freire M.G., Coutinho J.A.P., Palomar J. (2013). Composition and Structural Effects on the Adsorption of Ionic Liquids onto Activated Carbon. Environ. Sci. Process. Impacts.

[20] Szpecht A., Zajac A., Zielinski D., Maciejewski H., Smiglak M. (2019). Versatile Method for the Simultaneous Synthesis of Two Ionic Liquids, Otherwise Difficult to Obtain, with High Atom Economy. ChemistryOpen.

[21] Aparicio S., Atilhan M., Karadas F. (2010). Thermophysical Properties of Pure Ionic Liquids: Review of Present Situation. Ind. Eng. Chem. Res..

[22] Tiago G.A.O., Matias I.A.S., Ribeiro A.P.C., Martins L.M.D.R.S. (2020). Application of Ionic Liquids in Electrochemistry—Recent Advances. Molecules.

[23] Lockett V., Gustafson J., Ray W.J., Salah Y. (2018). Energy Storage Device, Ink for an Electrode of an Energy Storage Device and Method of Manufacturing an Energy Storage Device. TW Patent.

[24] Shi C., Ho C.C., Mackenzie J.D. (2021). Ionic Liquid Gel for Electrolyte, Method of and Ink for Making the Same, and Printed Batteries Including Such Ionic Liquid Gels and/or Electrolytes. Eur. Pat.

[25] Mackenzie J.D., Ho C., Yogeeswaran K., Roberts G., SHI C. (2020). Electrochemical Cells and Metal Salt-Based Electrolytes. U.S. Patent.

[26] Xiong W., Yin Z., Zhang X., Tu Z., Hu X., Wu Y. (2022). Ionic Liquids Endowed with Novel Hybrid Anions for Supercapacitors. ACS Omega.

[27] Livi S., Baudoux J., Gérard J.-F., Duchet-Rumeau J. (2022). Ionic Liquids: A Versatile Platform for the Design of a Multifunctional Epoxy Networks 2.0 Generation. Prog. Polym. Sci..

[28] Lv G., Jensen E., Shen C., Yang K., Evans C.M., Cahill D.G. (2021). Effect of Amine Hardener Molecular Structure on the Thermal Conductivity of Epoxy Resins. ACS Appl. Polym. Mater..

[29] Rahmathullah M.A.M., Jeyarajasingam A., Merritt B., VanLandingham M., McKnight S.H., Palmese G.R. (2009). Room Temperature Ionic Liquids as Thermally Latent Initiators for Polymerization of Epoxy Resins. Macromolecules.

[30] Jiang Z., Wang Q., Liu L., Zhang Y., Du F., Pang A. (2020). Dual-Functionalized Imidazolium Ionic Liquids as Curing Agents for Epoxy Resins. Ind. Eng. Chem. Res..

[31] Binks F.C., Cavalli G., Henningsen M., Howlin B.J., Hamerton I. (2018). Examining the Kinetics of the Thermal Polymerisation Behaviour of Epoxy Resins Initiated with a Series of 1-Ethyl-3-Methylimidazolium Based Ionic Liquids. Thermochim. Acta.

[32] Nguyen T.K.L., Livi S., Pruvost S., Soares B.G., Duchet-Rumeau J. (2014). Ionic Liquids as Reactive Additives for the Preparation and Modification of Epoxy Networks. J. Polym. Sci. Part A Polym. Chem..

[33] Maka H., Spychaj T., Pilawka R. (2012). Epoxy Resin/Ionic Liquid Systems: The Influence of Imidazolium Cation Size and Anion Type on Reactivity and Thermomechanical Properties. Ind. Eng. Chem. Res..

[34] Carvalho A.P.A., Santos D.F., Soares B.G. (2020). Epoxy/Imidazolium-based Ionic Liquid Systems: The Effect of the Hardener on the Curing Behavior, Thermal Stability, and Microwave Absorbing Properties. J. Appl. Polym. Sci..

[35] Nguyen T.K.L., Livi S., Soares B.G., Pruvost S., Duchet-Rumeau J., Gérard J.-F. (2016). Ionic Liquids: A New Route for the Design of Epoxy Networks. ACS Sustain. Chem. Eng..

[36] Xu Y.-J., Shi X.-H., Lu J.-H., Qi M., Guo D.-M., Chen L., Wang Y.-Z. (2020). Novel Phosphorus-Containing Imidazolium as Hardener for Epoxy Resin Aiming at Controllable Latent Curing Behavior and Flame Retardancy. Compos. Part B Eng..

[37] Varshneya A.K., Mauro J.C., Varshneya A.K., Mauro J.C. (2019). Chapter 2—Fundamentals of the Glassy State. Fundamentals of Inorganic Glasses.

[38] Gómez E., Calvar N., Domínguez Á., Gómez E., Calvar N., Domínguez Á. (2015). Thermal Behaviour of Pure Ionic Liquids.

[39] Shukla S.K., Mikkola J.-P., Zhang S. (2020). Melting Point of Ionic Liquids. Encyclopedia of Ionic Liquids.

[40] Dong K., Zhang S., Wang J. (2016). Understanding the Hydrogen Bonds in Ionic Liquids and Their Roles in Properties and Reactions. Chem. Commun..

[41] Domańska U., Żołek-Tryznowska Z., Królikowski M. (2007). Thermodynamic Phase Behavior of Ionic Liquids. J. Chem. Eng. Data.

[42] Siedlecka E., Czerwicka M., Stolte S., Stepnowski P. (2011). Stability of Ionic Liquids in Application Conditions. Curr. Org. Chem..

[43] Crosthwaite J.M., Muldoon M.J., Dixon J.K., Anderson J.L., Brennecke J.F. (2005). Phase Transition and Decomposition Temperatures, Heat Capacities and Viscosities of Pyridinium Ionic Liquids. J. Chem. Thermodyn..

[44] Fredlake C.P., Crosthwaite J.M., Hert D.G., Aki S.N.V.K., Brennecke J.F. (2004). Thermophysical Properties of Imidazolium-Based Ionic Liquids. J. Chem. Eng. Data.

[45] Pont A.-L., Marcilla R., De Meatza I., Grande H., Mecerreyes D. (2009). Pyrrolidinium-Based Polymeric Ionic Liquids as Mechanically and Electrochemically Stable Polymer Electrolytes. J. Power Source..

[46] Pitawela N.R., Shaw S.K. (2021). Imidazolium Triflate Ionic Liquids’ Capacitance–Potential Relationships and Transport Properties Affected by Cation Chain Lengths. ACS Meas. Sci. Au.

[47] Coustan L., Bélanger D. (2019). Electrochemical Activity of Platinum, Gold and Glassy Carbon Electrodes in Water-in-Salt Electrolyte. J. Electroanal. Chem..

[48] Sakaebe H., Matsumoto H., Tatsumi K. (2005). Discharge–Charge Properties of Li/LiCoO2 Cell Using Room Temperature Ionic Liquids (RTILs) Based on Quaternary Ammonium Cation—Effect of the Structure. J. Power Source..

