# Supplementary material for: Thermal and Electrochemical Properties of Ionic Liquids Bearing Allyl Group with Sulfonate-Based Anions—Application Potential in Epoxy Resin Curing Process
Source: Molecules. 2023 Jan 10;28(2):709. doi: 10.3390/molecules28020709 (PMC9864641; doi:10.3390/molecules28020709)
Supplement: Supplementary file 1 [file molecules-28-00709-s001.zip › molecules-2097523-supplementary.pdf]

Supplementary Materials

Thermal and Electrochemical Properties of Ionic Liquids Bearing Allyl Group with Sulfonate-Based Anions—Application Potential in Epoxy Resin Curing Process

Andrea Szpecht [1,*], Dawid Zielinski [1,2], Maciej Galinski [3] and Marcin Smiglak [1]

[1] Poznan Science and Technology Park, Adam Mickiewicz University Foundation, 61-612 Poznań, Poland
[2] Faculty of Chemistry, Adam Mickiewicz University, 61-614 Poznań, Poland
[3] Institute of Chemistry and Technical Electrochemistry, Poznan University of Technology, 60-965 Poznań, Poland
* Correspondence: andrea.szpecht@ppnt.poznan.pl

This document includes the synthetic procedure for compounds **1-4** from the manuscript. According to the synthetic procedure in the paper previously published by us [1], the following compounds were obtained:

S1 - 1-Allyl-3-methylimidazolium methylsulfate (**1**)
S2 - 1-Allyl-3-methylimidazolium methanesulfonate (**2**)
S3 - 1-Allyl-3-methylimidazolium triflate (**3**)
S4 - 1-Allyl-3-methylimidazolium tosylate (**4**)

S1 - 1-Allyl-3-methylimidazolium methylsulfate (**1**)

Yellow liquid; yield: 99%; purity: 98.4%.

^{1}H NMR (400 MHz, DMSO-d_6): δ = 9.11 (s, 1H), 7.73 (dt, ^{3}J = 7.6 Hz, ^{3}J = 1.8 Hz, 2H), 6.05 (ddt, ^{3}J = 16.3 Hz, ^{3}J = 10.3 Hz, ^{3}J = 6.0 Hz, 1H), 5.32 (ddd, ^{3}J = 6.7 Hz, ^{3}J = 4.1 Hz, ^{3}J = 1.3 Hz, 2H), 4.85 (d, ^{3}J = 6.0 Hz, 2H), 3.88 (s, ^{3}J = 7.4 Hz, 3H), 3.41 (s, 3H).

^{13}C {^{1}H} NMR (100 MHz, DMSO-d_6): δ = 137.35, 132.23, 124.23, 122.77, 120.66, 53.43, 51.20, 36.13.

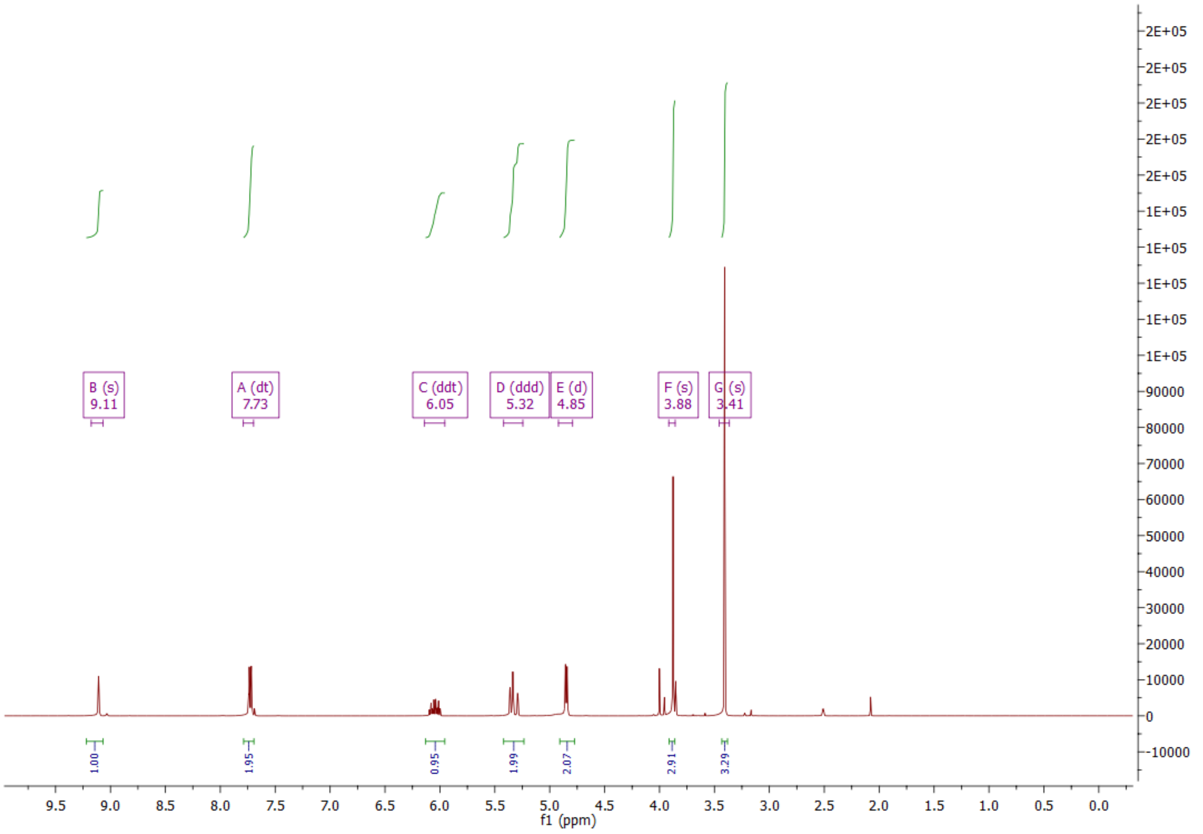

Figure S1a. ^{1}H NMR (400 MHz, DMSO-d_6) of **1.**

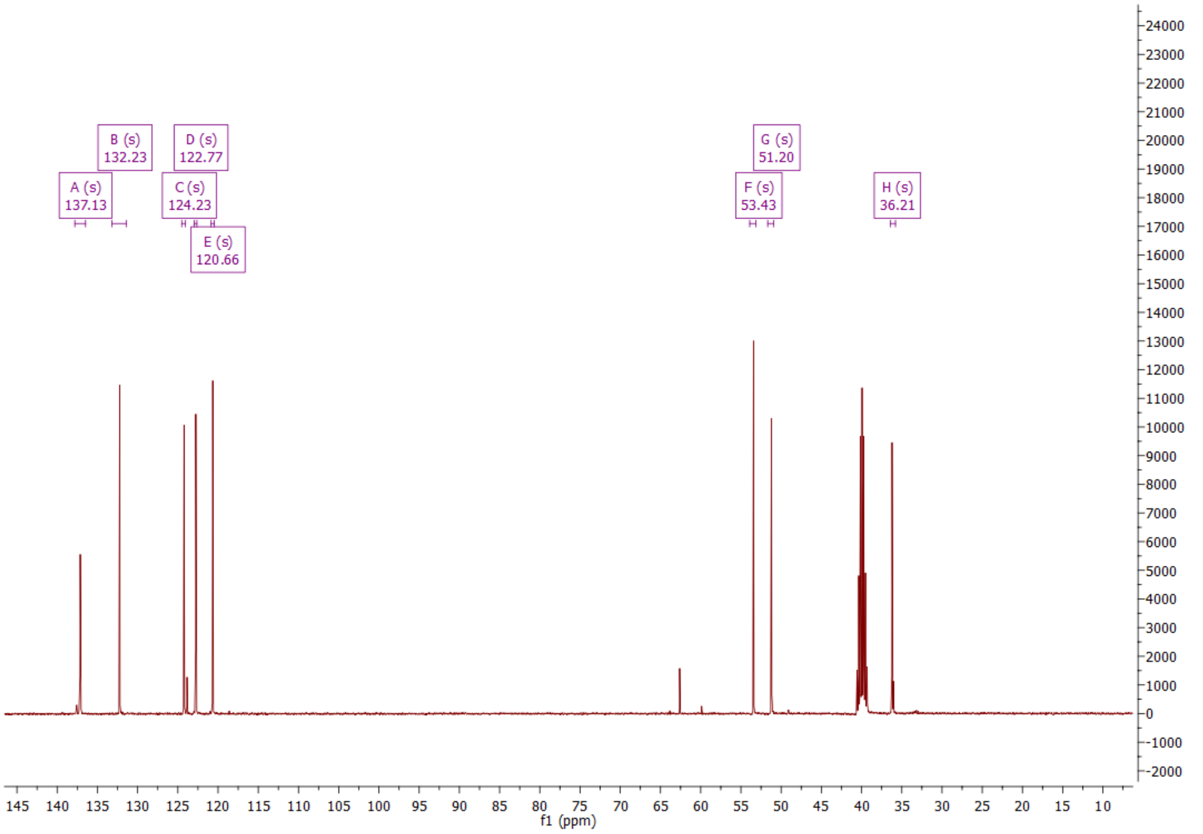

Figure S1b. ^{13}C {^{1}H} NMR (100 MHz, DMSO-d_6) of **1.**

S2 - 1-Allyl-3-methylimidazolium methanesulfonate (**2**)

Yellow liquid; yield: 99.2%; purity: 99.3%.
^{1}H NMR (400 MHz, $CDCl_3$): δ = 9.63 (s, 1H), 7.52 (s, 1H), 7.37 (s, 1H), 5.90 (ddt, ^{3}J = 16.5 Hz, ^{3}J = 10.2 Hz, ^{3}J = 6.3 Hz, 1H), 5.31 (t, ^{3}J = 13.3 Hz, 2H), 4.79 (d, ^{3}J = 6.2 Hz, 2H), 3.91 (s, 3H), 2.61 (s, 3H).
^{13}C {^{1}H} NMR (100 MHz, $CDCl_3$): δ = 137.63, 130. 22, 123.75, 122.02, 121.97, 51.66, 36.29.

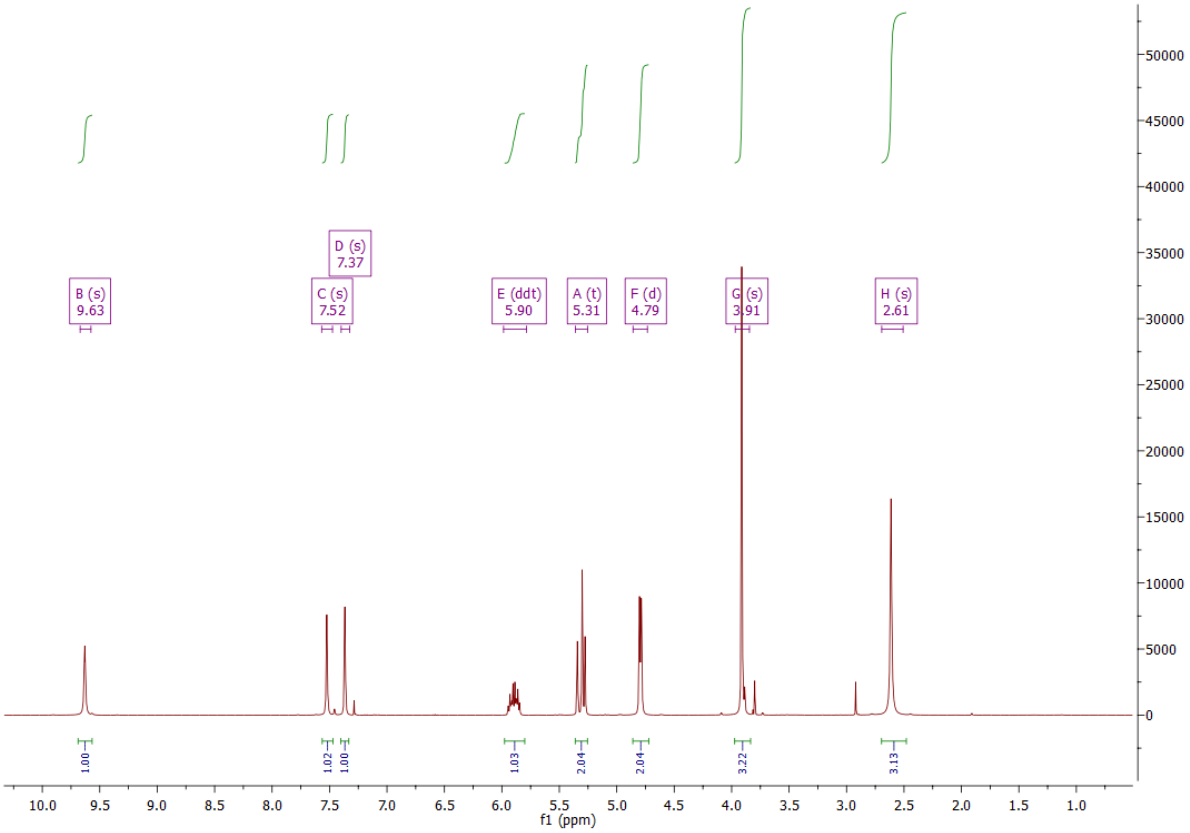

Figure S2a. ^{1}H NMR (400 MHz, $CDCl_3$) of **2.**

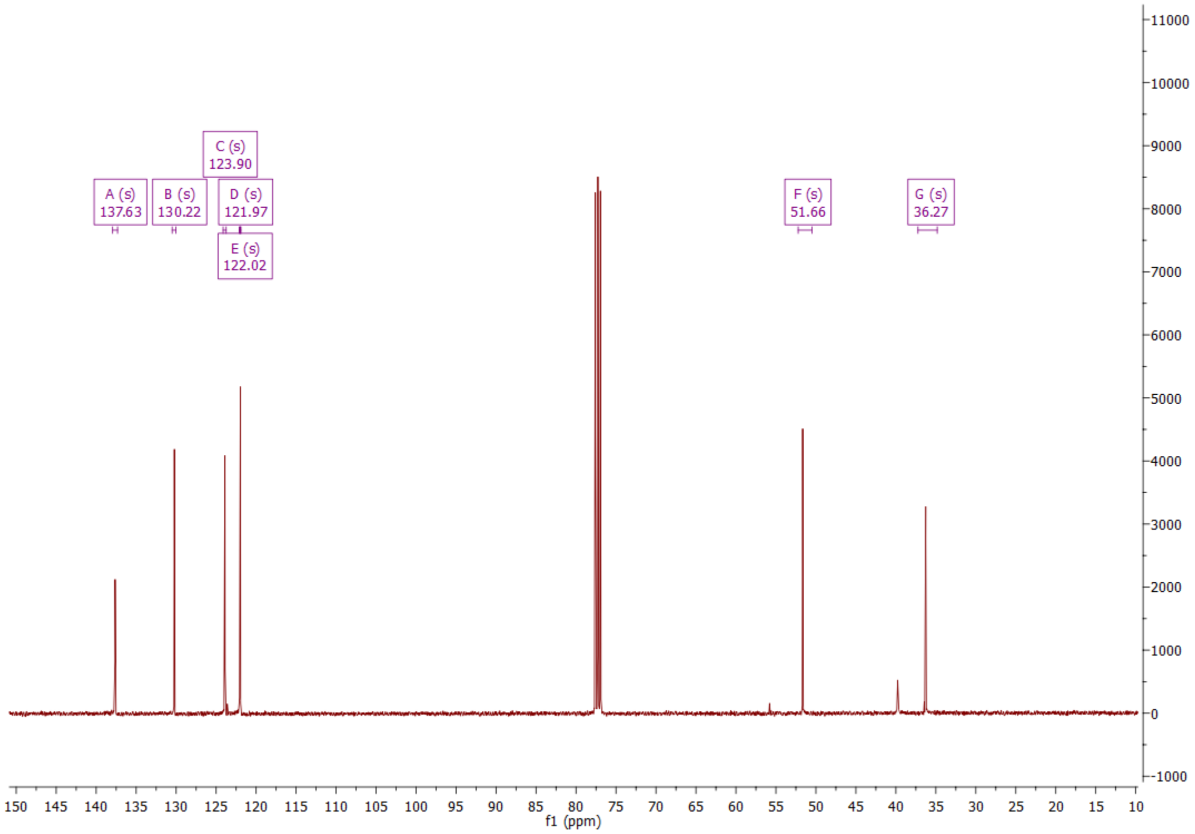

Figure S2b. ^{13}C {^{1}H} NMR (100 MHz, $CDCl_3$) of **2**.

S3 - 1-Allyl-3-methylimidazolium triflate (**3**)

Yellow liquid; yield: 99.5%; purity: 99.5%.

^{1}H NMR (400 MHz, DMSO-*d*$_6$): δ = 9.06 (s, 1H), 7.69 (dt, ^3J = 7.2 Hz, ^3J = 1.7 Hz, 2H), 6.03 (ddt, ^3J = 16.3 Hz, ^3J = 10.3 Hz, ^3J = 6.0 Hz, 1H), 5.35 (dt, ^3J = 5.0 Hz, ^3J = 2.5 Hz, 1H), 5.30 (dd, ^3J = 17.1 Hz, ^3J = 1.3 Hz, 1H), 4.83 (d, ^3J = 6.0 Hz, 2H), 3.86 (s, 3H)

^{19}F NMR (376 MHz, DMSO-*d*$_6$): δ = −77.87

^{13}C {^{1}H} NMR (100 MHz, DMSO-*d*$_6$): δ = 137.04, 132.06, 124.19, 122.72, 121.10 (q, ^3J = 322.1 Hz), 120.64, 51.24, 36.0.

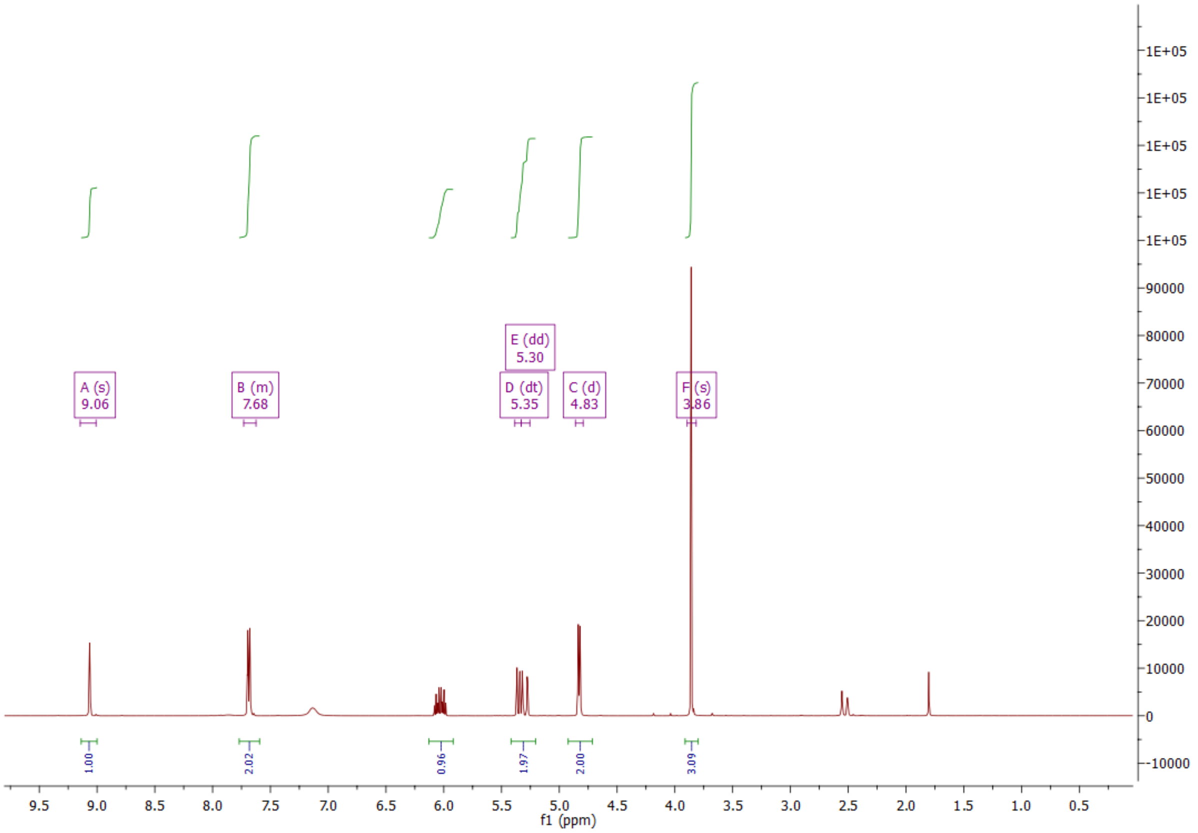

Figure S3a. ^{1}H NMR (400 MHz, DMSO-*d*$_6$) of **3.**

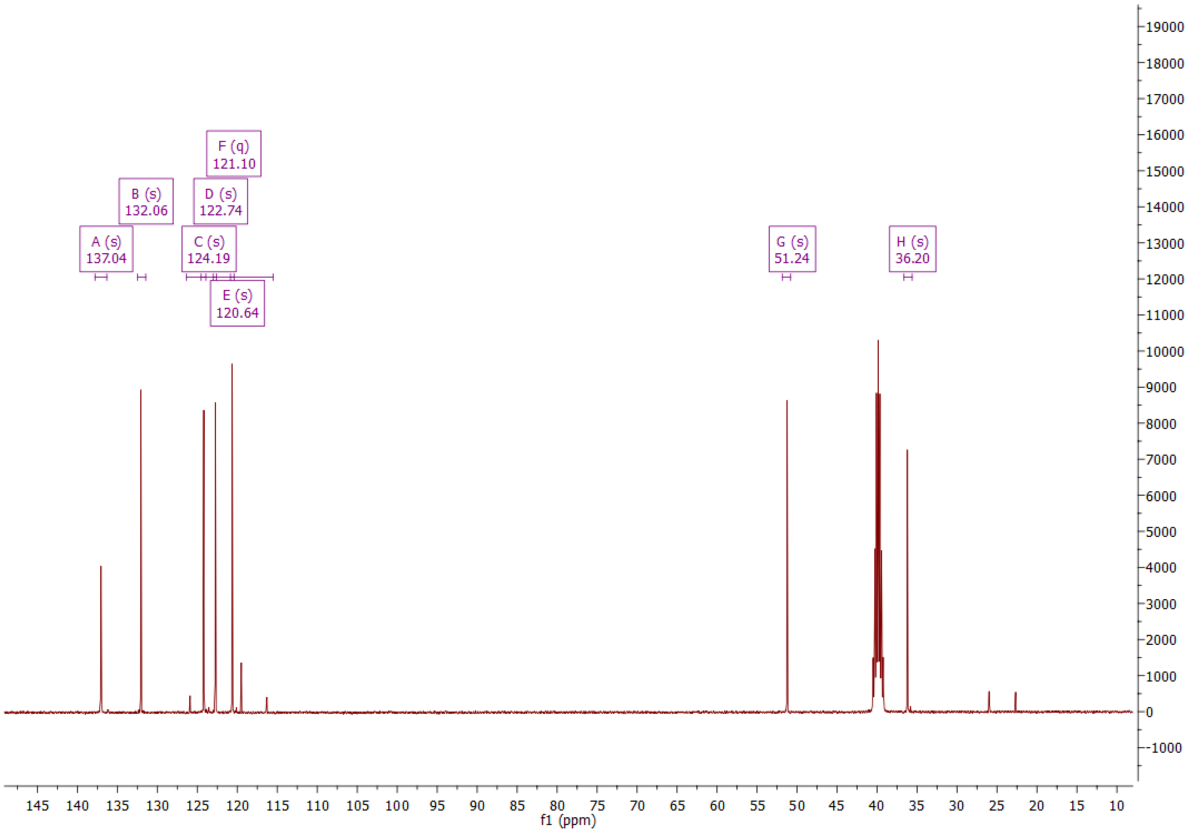

Figure S3b. ^{13}C {^{1}H} NMR (100 MHz, DMSO-*d*$_6$) of **3.**

S4 - 1-Allyl-3-methylimidazolium tosylate (**4**)

Yellow liquid; yield: 99.1%; purity: 99.2%.

^{1}H NMR (400 MHz, $CDCl_3$): δ = 9.50 (s, 1H), 7.66 (d, ^{3}J = 7.5 Hz, 2H), 7.46 (s, 1H), 7.29 (s, 1H), 7.07 (d, ^{3}J = 7.3 Hz, 2H), 5.82 (ddt, ^{3}J = 16.5 Hz, ^{3}J = 10.2 Hz, ^{3}J = 6.3 Hz, 1H), 5.25 (dd, ^{3}J = 13.6 Hz, ^{3}J = 7.2 Hz, 2H), 4.69 (d, ^{3}J = 6.2 Hz, 2H), 3.81 (s, 3H), 2.27 (s, 3H).

^{13}C {^{1}H} NMR (100 MHz, $CDCl_3$): δ = 139.39, 137.34, 130.18, 128.66, 125.70, 123.92, 121.98, 121.90, 51.61, 36.20, 21.22.

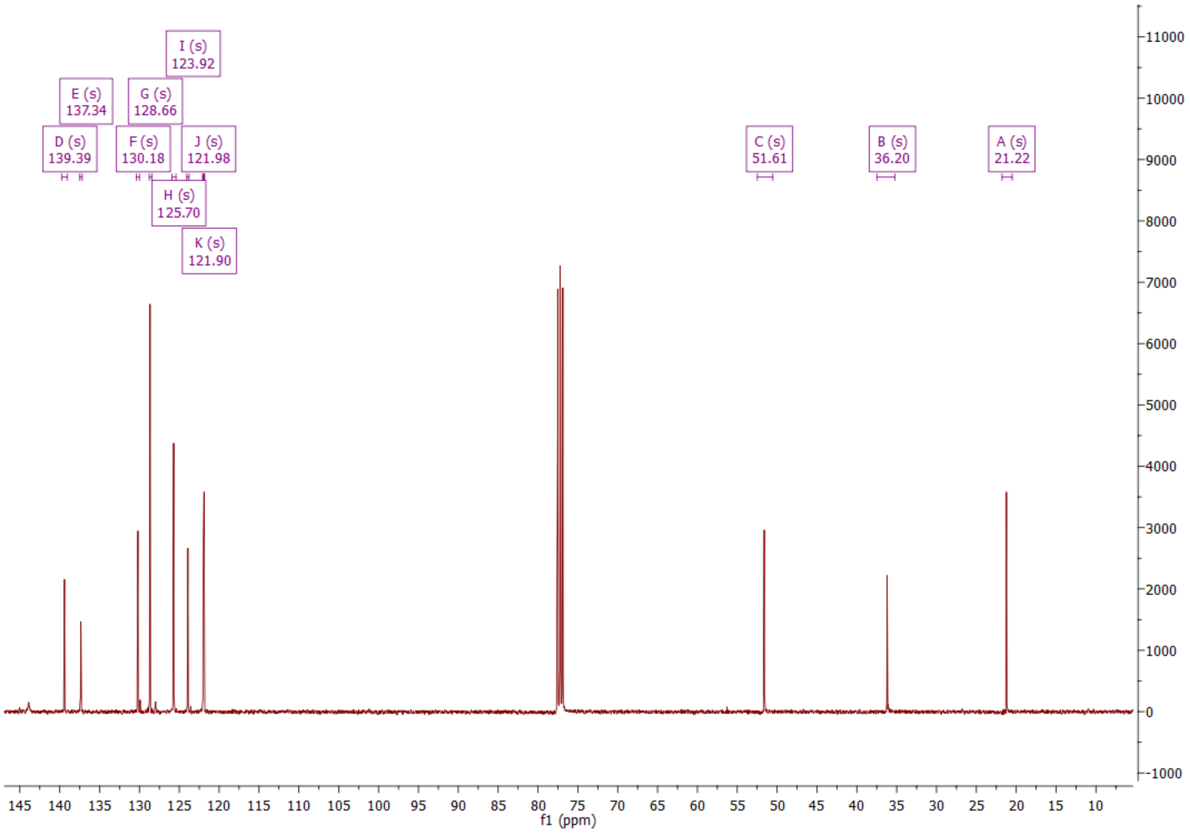

Figure S4a. ^{1}H NMR (400 MHz, $CDCl_3$) of **4.**

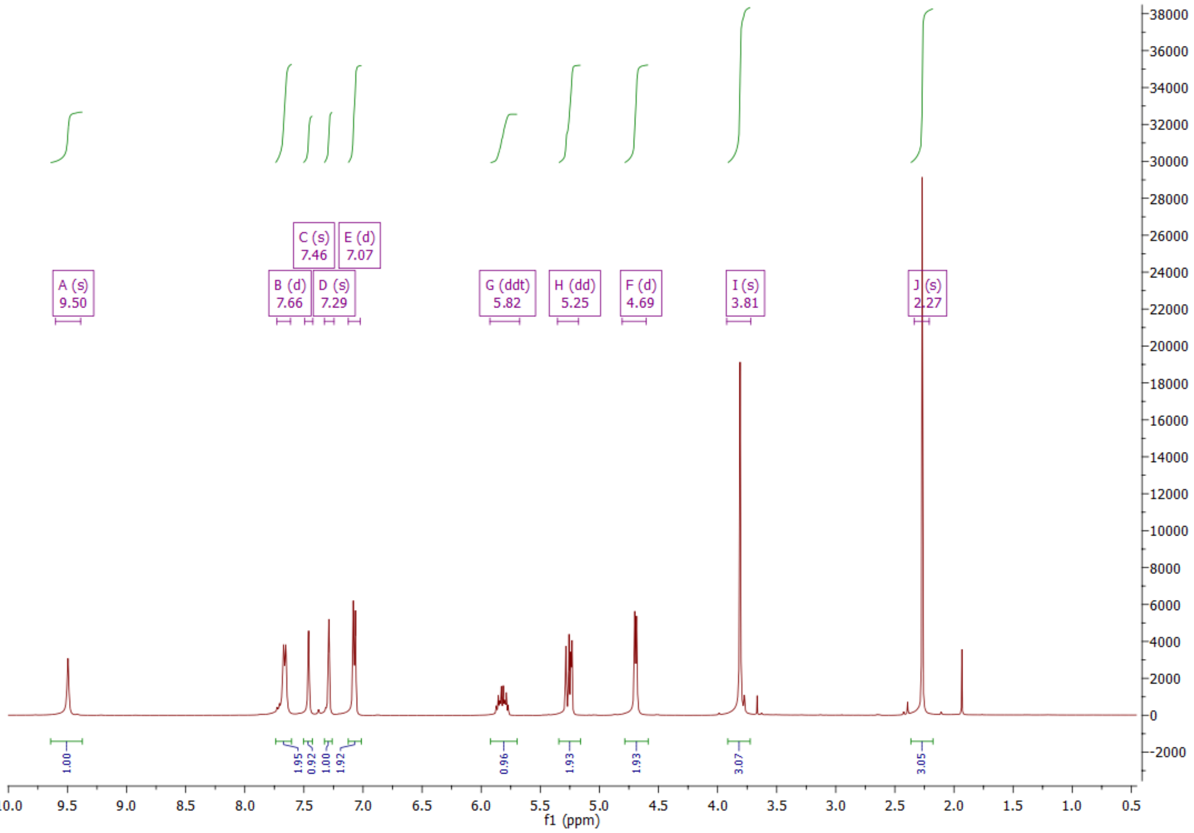

Figure S4b. ^{13}C {^{1}H} NMR (100 MHz, $CDCl_3$) of **4.**

References

1. Szpecht, A.; Zajac, A.; Zielinski, D.; Maciejewski, H.; Smiglak, M. Versatile Method for the Simultaneous Synthesis of Two Ionic Liquids, Otherwise Difficult to Obtain, with High Atom Economy. *ChemistryOpen* **2019**, *8*, 972–983, doi:10.1002/open.201900217.
